# War Disadvantages at King's College Hospital

**Published:** 1915-12-18

**Authors:** 


### WAR DISADVANTAGES AT KING'S COLLEGE HOSPITAL.

We have delayed publishing a descriptive report until sufficient time had elapsed to enable the new buildings at Denmark Hill to be completed and brought into full occupation and administrative smoothness. Although at the end of 1914 the whole of the new wards were completed and taken into use, with the exception of one in which the medical school was being temporarily accommodated pending the completion of the medical school buildings, the organisation and smooth working as a civil hospital were suddenly interrupted by an offer from the War Office to take over the whole of the buildings with the exception of the casualty department and at least four wards to be retained for the use of the civil population. The acceptance of this offer necessitated the closing of the out-patient department, and out-patients, except the relatively few referred from the casualty department and those sent for consultation by medical practitioners, or dealt with at the children's out-patient department, have been refused further treatment. The opening of the new buildings caused the numbers of out-patients admitted to treatment to increase from under 11,000 in 1913 to 31,500 in 1914. The position was extraordinary, the decision come to remarkable. Its effects upon the future popularity and financial prosperity of King's College Hospital in the coming years cannot fail to form a subject of great interest to hospital workers generally.

